# Gibberellin–Abscisic Acid Balances during Arbuscular Mycorrhiza Formation in Tomato

**DOI:** 10.3389/fpls.2016.01273

**Published:** 2016-08-23

**Authors:** José A. Martín-Rodríguez, Raúl Huertas, Tania Ho-Plágaro, Juan A. Ocampo, Veronika Turečková, Danuše Tarkowská, Jutta Ludwig-Müller, José M. García-Garrido

**Affiliations:** ^1^Department of Soil Microbiology and Symbiotic Systems, Estación Experimental del Zaidín, Consejo Superior de Investigaciones Científicas, GranadaSpain; ^2^Laboratory of Growth Regulators, Centre of the Region Haná for Biotechnological and Agricultural Research, Institute of Experimental Botany, Academy of Sciences of the Czech Republic, v.v.i., Palacký University, OlomoucCzech Republic; ^3^Institut für Botanik, Technische Universität Dresden, DresdenGermany

**Keywords:** arbuscular mycorrhiza, plant hormones, gibberellins, abscisic acid, symbiosis, tomato

## Abstract

Plant hormones have become appropriate candidates for driving functional plant mycorrhization programs, including the processes that regulate the formation of arbuscules in arbuscular mycorrhizal (AM) symbiosis. Here, we examine the role played by ABA/GA interactions regulating the formation of AM in tomato. We report differences in ABA and GA metabolism between control and mycorrhizal roots. Active synthesis and catabolism of ABA occur in AM roots. GAs level increases as a consequence of a symbiosis-induced mechanism that requires functional arbuscules which in turn is dependent on a functional ABA pathway. A negative interaction in their metabolism has been demonstrated. ABA attenuates GA-biosynthetic and increases GA-catabolic gene expression leading to a reduction in bioactive GAs. Vice versa, GA activated ABA catabolism mainly in mycorrhizal roots. The negative impact of GA_3_ on arbuscule abundance in wild-type plants is partially offset by treatment with ABA and the application of a GA biosynthesis inhibitor rescued the arbuscule abundance in the ABA-deficient *sitiens* mutant. These findings, coupled with the evidence that ABA application leads to reduce bioactive GA_1_, support the hypothesis that ABA could act modifying bioactive GA level to regulate AM. Taken together, our results suggest that these hormones perform essential functions and antagonize each other by oppositely regulating AM formation in tomato roots.

## Introduction

Several studies have shown that ABA and GAs, whose optimal balance is essential for normal plant development, interact antagonistically in numerous plant developmental processes (Weiss and Ori, 2007; Rodríguez-Gacio et al., 2009). ABA is a positive regulator of dormancy induction and its maintenance. On the other hand, while it is a negative regulator of germination, GAs release dormancy, promote germination and counteract the impact of ABA (Kucera et al., 2005). In citrus fruits, ABA increased and GA_20_ decreased under water stress conditions, whereas rehydration reduced ABA but increased GA_20_ (Mahouachi et al., 2005). In mature embryos, the inhibition of GA synthesis mimicked the effects of exogenous ABA by suppressing germination, acquiring anthocyanin pigments and by accumulating a variety of maturation-phase mRNAs (White et al., 2000). ABA and GAs also antagonistically regulate their own metabolic processes. Thus, low ABA level promotes GA biosynthesis (Seo et al., 2006) and vice versa (Oh et al., 2007). However, the precise molecular mechanism underlying ABA and GA antagonism is still unknown. A recent study points out that ABA-INSENSITIVE 4 (ABI4) is a central factor in GA/ABA homeostasis and suggests that ABA and GA antagonize each other by oppositely acting on ABI4 transcript and protein levels (Shu et al., 2016).

This antagonistic interaction between ABA and GAs could apply to their role in AM regulation. The AM is the most widespread mutualistic association in the plant kingdom. The efficiency of the symbiosis is mainly due to the development of an extensive and branched fungal hyphal network outside the roots that efficiently uptakes mineral nutrients (principally phosphorous) which are transferred to plant cortical cells by specialized intraradical, highly branched structures named arbuscules. In return the plant provides the fungus with carbon (Smith and Read, 2008). Fungal penetration and establishment in roots depend on a complex sequence of events and intracellular modifications that are dynamically regulated by the plant, probably according their physiological and developmental status that result from environmental conditions (Pozo et al., 2015).

Previous studies indicate that plant hormones become appropriate candidates for driving functional plant mycorrhization programs (Bonfante and Genre, 2010; Ludwig-Müller, 2010; Floss et al., 2013; Gutjahr, 2014). ABA is a sesquiterpenoid hormone, derived from carotenoids, which functions at multiple levels to regulate AM symbiosis. In this way in many plant species it has been observed as the concentration of ABA rises during the establishment of mycorrhization (Ludwig-Müller, 2010). Recent studies suggest a dose-dependent effect of ABA modulating the establishment of AM symbiosis in *Medicago truncatula* by promoting fungal colonization at low concentrations and its impairment at high concentrations (Charpentier et al., 2014). In tomato, the analysis of AM colonization in the ABA-deficient tomato mutant *sitiens*, deficient in functional enzyme activity at the final step in ABA biosynthesis, has demonstrated that ABA plays an important role in the development and functionality of arbuscules (Herrera-Medina et al., 2007; Martín-Rodríguez et al., 2010). In addition, ABA deficiency also results in the induction of ethylene production, which adversely affects mycorrhizal intensity (Herrera-Medina et al., 2007; Martín-Rodríguez et al., 2010, 2011). In addition, variations in the mycorrhization characteristics of wild-type and ABA-deficient tomato roots are accompanied by specific transcriptomic alterations associated with differences in the status of mycorrhization according to ABA content in roots (García-Garrido et al., 2010).

An opposite trend can be observed following the application of gibberellic acid (GA_3_), with a dose-dependent suppression of arbuscule formation being reported in plants treated with GA_3_ (El Ghachtouli et al., 1996; Foo et al., 2013; Martín-Rodríguez et al., 2015). Gibberellins are synthesized from carotenoid precursors by the action of GA 20-oxidases and 3β-hydroxylases. Several GA biosynthetic pathways with different hydroxylation patterns have been detected in various plant species (Hedden and Thomas, 2012), and GAs from the 13-hydroxylation biosynthetic pathway have been shown to be significantly more abundant in the roots of AM inoculated plants than in those of non-mycorrhizal plants (Shaul-Keinan et al., 2002; Martín-Rodríguez et al., 2015), while, in tomato, the higher levels of GAs in mycorrhizal roots correlate closely with increased gene expression associated with GA biosynthesis (Martín-Rodríguez et al., 2015). Furthermore, arbuscule formation is regulated by DELLA proteins in *M. truncatula* (Floss et al., 2013), rice (Yu et al., 2014), and pea (Foo et al., 2013). DELLA proteins are nuclear proteins that negatively regulate GA signaling. Conversely, GAs offset the effects of DELLA proteins by promoting DELLA protein destabilization (Silverstone et al., 2001). It has been suggested that DELLA is a positive regulator of arbuscule formation and also acts as a core connecting different signaling pathways activated during AM formation (Floss et al., 2013; Yu et al., 2014; Pimprikar et al., 2016).

Abscisic acid treatment has been shown to stabilize DELLA proteins in the presence of GAs (Achard et al., 2006), and both DELLA proteins and ABA act as positive regulators of arbuscule formation (reviewed by Gutjahr, 2014). The control of GA levels through a combination of biosynthesis and degradation in mycorrhizal roots could therefore partly depend on ABA, even more so when ABA and GAs antagonistically regulate their own metabolic processes (Seo et al., 2006; Oh et al., 2007). In this study, we have aimed to analyze the role played by ABA/GA imbalance regulating the formation of AM symbiosis in tomato plants. Using a combination of hormone analysis, gene expression, and application studies we demonstrate the antagonistic effects of both plant hormones on arbuscule abundance in roots. Being as ABA interacts with the expression pattern of metabolism-related GA genes in mycorrhizal plants, and the AM phenotype of low ABA mutant *sitiens* can be improved by GA biosynthesis inhibitor, we postulated that ABA may act in part via modifying bioactive GA level to regulate AM. Furthermore, an antagonistic effect of GA_3_ applications on endogenous ABA accumulation was observed, suggesting that the imbalance in the ABA/GA ratio is capable of reducing arbuscule abundance in these mutants. We therefore conclude that the balance between ABA and GAs is essential for AM formation in tomato roots.

## Materials and Methods

### Plant Growth and AM Inoculation

*Solanum lycopersicum* L. (Mill.) cv. Moneymaker (accession LA0575), the ABA-deficient mutant *sitiens* (Taylor et al., 1988; accession LA3283) and its wild-type background cv. Rheinlands Ruhm (Accession LA0535), the GA-constitutive response mutant *procera* (Bassel et al., 2008) and its isogenic wild-type Ailsa Craig were used. Seed were obtained from the Tomato Genetics Resource Centre (TGRC) at the University of California, Davis, CA, USA. Tomato seed sterilization, AM fungi inoculation and plant growth were carried out according to the techniques described by Herrera-Medina et al. (2007). Plant growth and treatments were carried out in a growth chamber (16:8 h, 24:19°C, day:night cycle; relative humidity 50%). Inoculation with *Rhizophagus irregularis* (DAOM 197198; Schüßler and Walker, 2010) was carried out in 200 mL pots. Each seedling was grown in a separate pot and inoculated with a piece of monoxenic culture in a Gel-Gro medium (ICN Biochemicals, Aurora, OH, USA) containing 50 *R. irregularis* spores and infected carrot roots. The monoxenic culture (*R. irregularis* and carrot roots) was produced according to the method described by Chabot et al. (1992). In the non-inoculated treatment, a piece of Gel-Gro medium containing only uninfected carrot roots was applied to the plants.

One week after planting in pots and weekly thereafter, 20 mL of a modified Long Ashton nutrient solution containing 0.325 mM Pi was added to prevent mycorrhizal inhibition by excess phosphorous (Hewitt, 1966). Non-mycorrhizal control plants received complete Long Ashton nutrient solution (1.3 mM Pi). Plants were harvested at 50 days after inoculation, and the root system was washed, rinsed several times with sterilized distilled water, weighed and cut into pieces of about 1 cm pieces from each root system were mixed to obtain a homogenate mixture of the whole root system. Representative portions of this mixture were handled and used for different measurements according to the nature of the experiment. Root pieces for tripan blue staining were directly processed. Roots for RNA extractions were stored at -80°C until use, and root pieces for hormone quantification were frozen and lyophilised. In each experiment, five independent plants were analyzed per treatment. Each plant was considered as a biological replicate. *Sitiens* plants were sprayed daily with water to prevent wilting.

### Estimation of Root Colonization

The non-vital trypan blue histochemical staining procedure was used according to the Phillips and Hayman (1970) method. Stained roots were observed under a light microscope, and the intensity of root cortex colonization by the AM fungus was determined according to the procedure described by Trouvelot et al. (1986) using MYCOCALC software^1^. The parameters measured were frequency of colonization (%F), intensity of colonization (%M) and arbuscule abundance in the whole root (%A) as well as arbuscule abundance in mycorrhizal root fragments (%a). At least three microscope slides were analyzed per root system, with each slide containing 30 1 cm root pieces.

The percentage of arbuscules at three distinct morphological stages of arbuscule formation was calculated according previous studies (Herrera-Medina et al., 2007). Class A are arbuscules in formation (or degradation) with no fine branches partially occupying the plant cell; class B, arbuscules with at least some fine branches occupying almost all of the plant cell; class C, arbuscules with many branches occupying the whole plant cell. A brief description of the evaluation of arbuscule morphology was shown in Supplementary Figure S1.

### RNA Extractions and Gene Expression

For the RT-PCR experiments, total RNA was isolated from 0.5 g sample taken from the root of each plant. Total RNA was isolated from the roots stored at -80°C using the RNeasy Plant Mini Kit (Qiagen, Valencia, CA, USA) following the manufacturer’s instructions.

cDNAs were obtained from 1 μg of total DNAse-treated RNA in a 20 μL reaction volume using the iScriptTM cDNA synthesis kit following the supplier’s protocol (Bio-Rad, Hercules, CA, USA). qRT-PCR was carried out to measure the transcript abundance of the elongation factor 1α (*GinEF*) gene of *R. irregularis*, as well as *SlPt4* and GA- and ABA-pathway related tomato genes. Primer names and corresponding sequences (Supplementary Table S1) were previously published. qRT-PCR was carried out using an iCycler apparatus (Bio-Rad, Hercules, CA, USA). Each 20 μL PCR reaction contained 1 μL of diluted cDNA (1:10), 10 μL 2x SYBR Green Supermix (Bio-Rad, Hercules, CA, USA) and 200 nM of each primer using a 96-well plate. The PCR program consisted of a 3 min incubation at 95°C, followed by 35 cycles of 30 s at 95°C, 30 s at 58–63°C, and 30 s at 72°C. The specificity of the PCR amplification procedure was checked using a melting curve after the final PCR cycle (70 steps of 30 s from 60 to 95°C at a heating rate of 0.5°C). Experiments were carried out on three biological replicates, and the threshold cycle (Ct) was determined in triplicate. The relative transcription levels were calculated by using the 2^-ΔΔCt^ method (Livak and Schmittgen, 2001). The Ct values of all genes were normalized to the Ct value of the *LeEF-1* (accession number X14449) housekeeping gene.

The qPCR data for each gene were shown as relative expression with respect to the control treatment (“reference treatment”) to which it was assigned an expression value of 1. The reference treatment generally corresponded to the non-AM inoculated treatment. When comparing different genes in a single experiment, qPCR data were showed as *M*-value (the *M*-value refers to log2 of the relative expression level).

### Chemical Treatments

The tomato plants were treated in soil with ABA (Sigma), gibberellic acid (GA_3_; Panreac, Barcelona, Spain), and prohexadione-calcium (PrCa; BASF). The PrCa is a late stage GA biosynthetic inhibitor that blocks 3β-hydroxylation and also prevents catabolism of GA_1_ to inactive GA_8_ by blocking 2β-hydroxylation (Brown et al., 1997). The solutions were prepared by dilution from a stock. Stock solutions contained 1 mM ABA, 100 μM GA_3_ and 1 mM PrCa in 1% ethanol. A 0.1% ethanol solution was used for the control treatments to compensate the ethanol content present in the hormonal solutions applied. The final ABA, GA_3_, and PrCa concentrations were in the range of concentrations used in previous studies in which its effect on AM formation were determined (Martín-Rodríguez et al., 2010; Martín-Rodríguez et al., 2015). Twenty ml of the corresponding diluted solution were applied twice a week to each 200 mL pot containing one tomato plant. The first application was carried out 1 week after AM fungal inoculation.

### Hormone Quantification

Abscisic acid quantification was repeated three times with each biological replicates. Freeze-dried roots (equivalent to a minimum of 100 mg fresh weight of root material) were homogenized and extracted for 1 h in 1 mL cold methanol/water/acetic acid (80/19/1, v/v). Internal standard mixtures, containing 20 pmoL of each of (-)-7′,7′,7′-2H_3_-phaseic acid; (-)-7′,7′,7′-2H_3_-dihydrophaseic acid; (-)-8′,8′,8′-2H_3_-neophaseic acid; (+)-4,5,8′,8′,8′-2H_5_-ABA-GE; (-)-5,8′,8′,8′-2H_4_-7′-OH-ABA and (+)-3′,5′,5′,7′,7′,7′-2H_6_-ABA were added to the samples. The homogenates were centrifuged (21000 × *g*, 10 min, 4°C) and the pellets were then re-extracted in 0.5 mL extraction solvent for 30 min. The supernatants were dried under vacuum. Extracts were dissolved in 100 μL 99% methanol:1% acetic acid (v/v) topped up to 1 mL with 99% water:1% acetic acid (v/v), purified by solid-phase extraction on an Oasis^®^ HLB cartridges (60 mg, 3 mL, Waters, Milford, MA, USA) and evaporated to dryness in a Speed-Vac (UniEquip). Subsequently the evaporated samples were methylated, purified by ABA-specific immunoaffinity extraction (Hradecká et al., 2007), and analyzed by UPLC-ESI(+)-MS/MS (Turečková et al., 2009).

Samples were analyzed for GA content according to Urbanová et al. (2013) with modifications (Martín-Rodríguez et al., 2015). Tomato root samples (10 mg freeze-dried dry weight) were homogenized using a MM 301 vibration mill (Retsch GmbH and Co. KG, Haan, Germany) in 2 mL-Eppendorf tubes with 1 mL 80% acetonitrile containing 5% formic acid after adding internal standard mixture containing 50 pmol each of ^2^H_2_-labeled GAs ([^2^H_2_]GA_1_, [^2^H_2_]GA_3_, [^2^H_2_]GA_4_, [^2^H_2_]GA_5_, [^2^H_2_]GA_6_, [^2^H_2_]GA_7_, [^2^H_2_]GA_8_, [^2^H_2_]GA_9_, [^2^H_2_]GA_12_, [^2^H_2_]GA_12ald_, [^2^H_2_]GA_15_, [^2^H_2_]GA_19_, [^2^H_2_]GA_20_, [^2^H_2_]GA_24_, [^2^H_2_]GA_29_, [^2^H_2_]GA_34_, [^2^H_2_]GA_44_, [^2^H_2_]GA_51_, and [^2^H_2_]GA_53_; OlChemIm, Olomouc, Czech Republic). The tubes were then placed in a fridge (4°C) and extracted overnight by stirring at 15 rpm using a bench top laboratory rotator Stuart SB 3^2^. The homogenates were centrifuged at 19000 rpm for 10 min at 4°C using a Beckman Avanti^TM^ 30 centrifuge^3^. Supernatants were further purified using mixed mode anion exchange cartridges^4^ and analyzed by ultra-high performance chromatography (Acquity UPLC^TM^ System; Waters) coupled to a triple-stage quadrupole mass spectrometer (Xevo^®^ TQ MS, Waters MS Technologies, Manchester, UK) equipped with an electrospray (ESI) interface. Gibberellins were detected in multiple-reaction monitoring (MRM) mode based on the transition of the precursor ion [M-H]- to the appropriate product ion. Data were obtained and processed by Masslynx 4.1 software (Waters) and GA levels were calculated using the standard isotope-dilution method (Rittenberg and Foster, 1940).

### Statistical Analysis

Data were analyzed by general linear model analysis of variance (ANOVA), with subsequent comparison between means using Duncan’s multiple range test (*P* = 0.05). Correlation and regression analysis was done using Microsoft Excel 2010 software.

## Results and Discussion

Plant hormones mostly work in combination, with hormonal balance being critical for short- and long-term responses. In several plant species has now been established that ABA plays a largely positive role in AM, while GAs acting through DELLAs have a negative role. Most evidence derives from pharmacological or genetic approaches through the analysis of plants with altered hormone biosynthesis or signaling (Pozo et al., 2015). In this study, we attempt to demonstrate that the role played by ABA in AM formation could, at least in part, be attributable to antagonistic interactions with GAs and to explore the possible ways in which ABA and GAs interact to control AM.

### Metabolism and Distribution of ABA in Arbuscular Mycorrhizal Tomato Roots

Our results show that differences in ABA metabolism between control and mycorrhizal roots exist (**Table 1**). In tomato roots, the main ABA metabolism pathway appears to go through 8′- hydroxylation (resulting in PA which is further reduced to DPA) and conjugation (resulting in ABA-GE). Secondary catabolism pathways like 7′- and 9′-hydroxylation (resulting in 7′-OH ABA and neoPA) are also present. Mycorrhizal roots showed a slight increase in free ABA and possess smaller amounts of ABA catabolites and much less ABA-GE than that in non-mycorrhizal roots (**Table 1**). The presence of ABA catabolites suggests that bioactive ABA was previously biosynthesized in the tissue and then rapidly metabolized. A comparative analysis of ABA metabolite distribution in non-inoculated and inoculated plant roots it shown in Supplementary Figure S2. Significantly, ABA-GE metabolite constitutes 80% of total ABA in control roots, and only 43% in colonized roots, while free ABA accounts 20% of total ABA in mycorrhizal roots and only 7% in the controls.

**Table 1:** Abscisic acid metabolites content (ng/g DW) in tomato roots.

	Control (C)	Mycorrhizal (M)	Ratio M/C
Free ABA	99 ± 2.8^a^	137 ± 19.2^b^	1.38
ABA-GE	1598 ± 284.7^a^	311 ± 27.7^b^	0.19
DPA	276 ± 41^a^	228 ± 80^a^	0.82
PA	59 ± 2.13^a^	9 ± 0.3^b^	0.15
7′OH-ABA	ND	8 ± 3.3^a^	≥8
neo-PA	13 ± 3.1^a^	5 ± 0.9^b^	0.38

*Levels of ABA, ABA-GE, DPA, PA, 7′ OH-ABA and neo-PA were measured by UPLC-ESI(+)-MS/MS from roots of non-mycorrhizal (control) and mycorrhizal plants 50 days after inoculation with *Rhizophagus irregularis*. Values correspond to mean ± SE (*n* = 5). For each compound values with same letters do not significantly differ (*P* = 0.05) according to Duncan’s multiple range test.*

**FIGURE 1:**
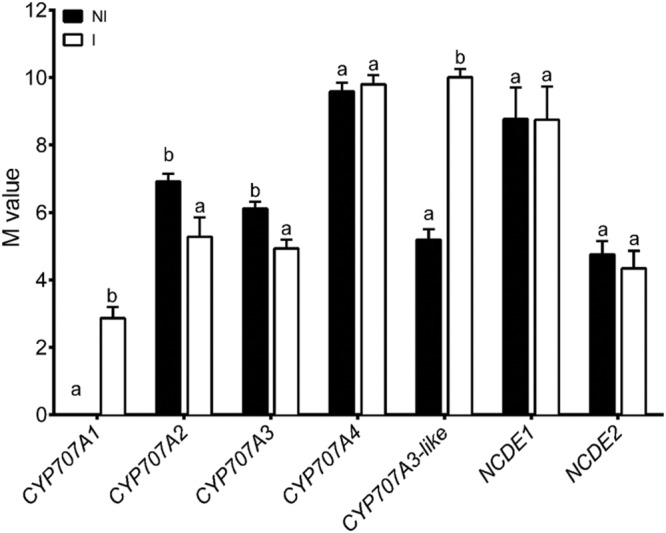
**Abscisic acid metabolism-related gene expression in roots of Moneymaker tomato plants non-colonized (NI) or colonized with *R. irregularis* (I).** Gene expression was measured by qPCR 50 days after inoculation. qPCR data represent the expression of target genes as *M*-value with respect the expression of *CYP707A1* in non-colonized plants (the biological replicate with lower expression) in which the expression was designated to be *M* = 0 and all other samples were expressed relative to it. Values correspond to mean ± SE (*n* = 3). For each gene, bars with same letters are not significantly different (*P* = 0.05) according to Duncan’s multiple range test.

The concentration of ABA is regulated by catabolism as well as synthesis. ABA hydroxylation is catalyzed by cytochrome P450 mono-oxygenases (CYP707A) and, in tomato, we identified five cytochrome P450 proteins that contain the highly conserved cysteine residue (within the PFGNGTHS**C**PG motif), which is the putative heme-iron ligand, common to all P450s and essential for catalytic activity (Kushiro et al., 2004), and putatively encode ABA hydroxylases (Supplementary Table S1). Additionally, we detected two tomato NCED that catalyze the major rate-limiting step and point of regulation in ABA biosynthesis (Supplementary Table S1). Real-time PCR analysis revealed differences in gene expression between control and inoculated tomato roots. *SlNCED1* and *SlNCED2* are equally expressed in mycorrhizal and non-mycorrhizal roots, although *SlNCED1* was expressed approximately 30 times more than *SlNCED2* (**Figure 1**). Gene expression data in combination with the results of ABA measurements suggest that some alterations in ABA catabolites could be due to differential regulation of ABA-hydroxylation related genes. Thus, four genes encoding putative ABA hydroxylases are responsive to colonization, two of which (*SlCYP707A1* and *SlCYP707A3*-like) are up-regulated and two (*SlCYP707A2* and *SlCYP707A3*) repressed in mycorrhizal roots (**Figure 1**). These data are fully in line with these provided by of Fiorilli et al. (2009), who suggest that active synthesis and catabolism of ABA occur in mycorrhizal roots and provide evidence of the presence of a *CYP707A* gene that is specifically expressed in arbuscule-containing cells. All the data related to the study of ABA during AM formation support the hypothesis that a balance between the biosynthesis and catabolism of ABA is crucial for the differentiation of arbuscules in tomato (Herrera-Medina et al., 2007; Fiorilli et al., 2009; García-Garrido et al., 2010; this study).

A role for ABA in the mechanisms by which AM symbiosis influences water stress has recently been suggested (Ludwig-Müller, 2010; Ruiz-Lozano et al., 2016). Recent results showed a steady increase in ABA content in tomato roots from non-AM plants as a consequence of water stress, reaching the maximum ABA levels under severe stress. A similar trend as for non-AM plants was observed for AM plants, even though mycorrhizal tomato plants showed a significant lower level of ABA in roots compared to non-AM plants (Chitarra et al., 2016; Ruiz-Lozano et al., 2016). In plants, two pathways promote free ABA accumulation: NCED-mediated *de novo* synthesis and beta-glucosidase-mediated hydroxylation (Lee et al., 2006). It was demonstrated that β-glucosidases from *Arabidopsis* can catalyze the release of ABA-GE into free ABA for involvement in stomatal movement, early seed germination, and abiotic stress (Lee et al., 2006). This release of ABA from ABA-GE pools is an important mechanism for regulating ABA levels. Therefore, it is conceivable that the differences in the accumulation of free ABA under water stress between non-mycorrhizal and mycorrhizal tomato plants (Chitarra et al., 2016; Ruiz-Lozano et al., 2016) could be a consequence of the lower ABA-GE content in mycorrhizal plants, as it has been demonstrated here.

### GA Metabolism in Low ABA Mutant

As previously shown (Martín-Rodríguez et al., 2015), GA metabolism from the 13-hydroxylation pathway sharply increased in tomato mycorrhizal roots. To test if ABA interferes with the GA metabolism upon mycorrhization, we analyzed the differences in GA concentration and GA-related gene expression between wild-type mycorrhizal plants and ABA-deficient mutant plants which have shown mycorrhizal impairment (Herrera-Medina et al., 2007).

**FIGURE 2:**
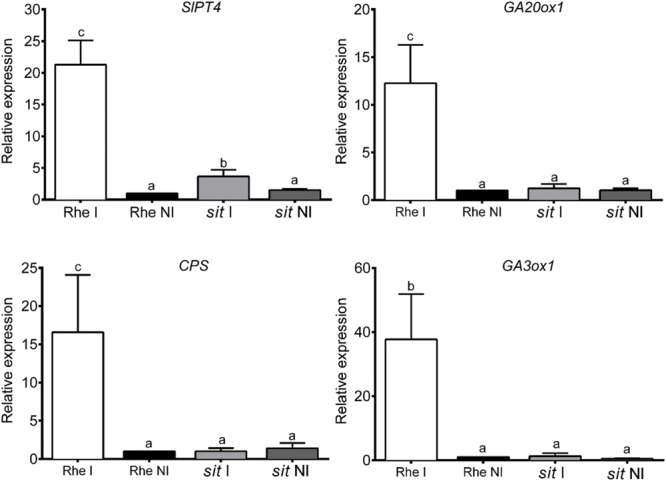
***SlPT4* and GA metabolism-related gene expression in roots of Rheinland Ruhm (Rhe) and *sitiens* (sit) tomato plants non-colonized (NI) and colonized (I) with *R. irregularis*.** After 1 week of transplanting, plants were inoculated with *R. irregularis* and gene expression was measured by qPCR 50 days after inoculation. qPCR data represent the relative expression of target genes in plants with respect to expression in Rheinland Ruhm non-colonized plants in which expression was designated as 1. Values correspond to mean ± SE (*n* = 3). For each gene, bars with same letters are not significantly different (*P* = 0.05) according to Duncan’s multiple range test.

Arbuscular mycorrhiza functionality was quantified as *SlPT4* gene expression as *SlPT4* encodes an AM-specific phosphate transporter protein whose transcript levels are a measure of arbuscule functionality (Balestrini et al., 2007). Fungal colonization significantly increased *SlPT4* gene expression in wild-type plants, while only a slight increase in transcript accumulation was detected in mycorrhizal *sitiens* plants (**Figure 2**). These data reflect the impairment of ABA-deficient plants in terms of establishing functional mycorrhization.

The expression pattern of GA biosynthesis-related genes coding for CPS, GA 20-oxidase 1 (GA20ox1) and GA 3-oxidase 1 (GA3ox1) was analyzed. For all three genes, expression levels were higher in the roots of wild-type mycorrhizal plants than in wild-type non-mycorrhizal plants. Gene expression levels in the roots of both mycorrhizal and non-mycorrhizal *sitiens* plants were similar to those found in wild-type non-mycorrhizal plants (**Figure 2**).

Concentrations of several endogenous GAs were measured in the roots of wild-type and *sitiens* plants relative to their mycorrhization capacity (Supplementary Figure S3). Colonization of wild-type tomatoes caused an increase in active GA_1_ and GA_3_ compounds, derived from the 13-hydroxylation pathway, relative to those in the non-mycorrhizal controls, while a decrease in the GA_5_, a precursor of GA_3_, was observed (Supplementary Figure S3). In the case of *sitiens* plants, no major changes were observed in GA content associated with either, ABA deficiency or mycorrhization. Only GA_19_ content increased following colonization, while non-mycorrhizal and mycorrhizal *sitiens* plants showed similar GA_44_ and GA_5_ levels than wild-type mycorrhizal plants (Supplementary Figure S3).

In conclusion, the increases of GAs were either absent or less pronounced in the impaired mycorrhizal ABA-deficient *sitiens* mutant than in wild-type plants, with only a slight increase in the expression of GA catabolism and GA responsive genes being associated with ABA-deficiency. These results suggest that a functional and well-established colonization is necessary for the expression of GA-related genes in mycorrhizal roots. They also clearly show that the increase in GA levels in mycorrhizal roots is a consequence of a symbiosis-induced mechanism that requires well-established colonization and functional arbuscules which in turn is dependent on a functional ABA pathway (Herrera-Medina et al., 2007; Martín-Rodríguez et al., 2011).

### ABA Influences GAs and then Influence AM Formation

**FIGURE 3:**
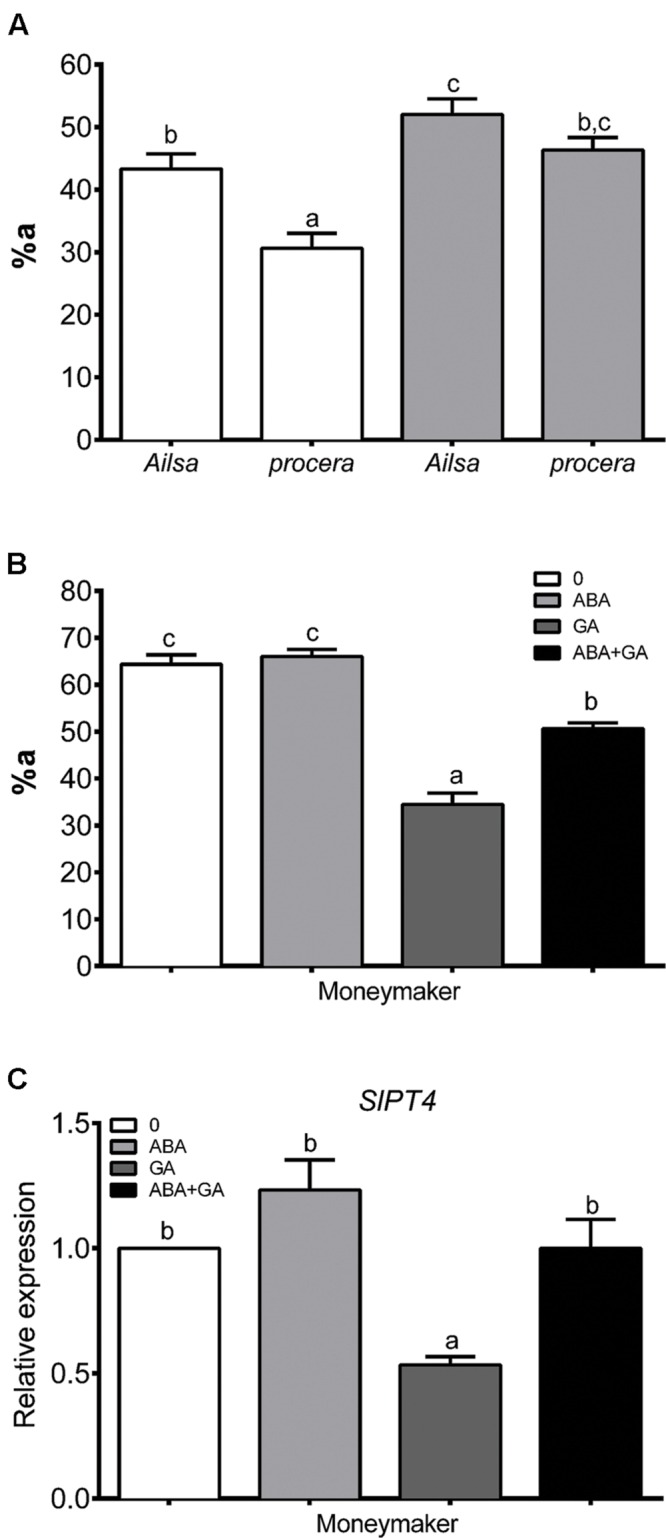
**Arbuscular abundance in mycorrhizal roots of non-treated Ailsa Craig and *procera* plants **(A)** and arbuscular abundance **(B)** and *SlPT4* gene expression **(C)** in Moneymaker tomato plants after ABA and GA applications.** After 1 week of transplanting and inoculation with *R. irregularis*, plants were treated with ABA (75 μM) and GA3 (5 μM). Solutions were applied to soil twice per week and mycorrhization was measured 50 days after inoculation. Arbuscular abundance (a%) in mycorrhizal zones of the root was determined using MYCOCALC software. qPCR data represent the expression of *SlPT4* gene in treated plants with respect to expression in non-treated plants in which expression was designated as 1. Values correspond to mean ± SE (*n* = 3). Bars with same letters do not significantly differ (*P* = 0.05) according to Duncan’s multiple range test.

A set of experiments were carried out in order to establish a link between ABA/GA imbalance and regulation of AM formation. Firstly, arbuscule abundance (%a) in mycorrhizal root fragments was determined in Ailsa Craig wild-type and *procera* (a GA-constitutive response tomato mutant) plants treated with ABA (**Figure 3A**). Non-treated *procera* plants showed lower arbuscule abundance levels in roots than wild-type plants. However, ABA treatment rescued the %a values for *procera* roots, bringing them into the same range as those recorded for wild-type plant roots. ABA treatment did not increase %a in wild-type plants (**Figure 3A**).

Secondly, arbuscule abundance (%a) in the mycorrhizal root fragments of wild-type plants treated with ABA, GA_3_, and ABA+GA_3_ was determined (**Figure 3B**). Although, GA_3_ treatment negatively affected %a in wild-type mycorrhizal tomato roots, this parameter was shown to be partially rescued when ABA was combined with the application of GA_3_, reaching 75% as compared to the value observed for non-treated plants (**Figure 3B**). Fungal colonization was also quantified as *SlPT4* gene expression. As with %a, GA_3_ treatment decreased *SlPT4* gene expression in mycorrhizal roots, while ABA rescued the negative impact of GA when both compounds were applied together (**Figure 3C**).

The negative effect on arbuscule abundance due to the applications of GA_3_ to wild-type plants, and the depletion of arbuscules in *procera* mutant plants carrying a mutation in the gene encoding SlDELLA, a repressor in the GA-signaling pathway (Bassel et al., 2008), were both partly offset by the application of ABA. The fact that ABA can rescue arbuscules in *procera* mutant plants lacking a functional DELLA protein means that the effect of ABA applications should not be attributed to DELLA stabilization which is the main target of ABA/GA interaction and suggests that ABA may act downstream and independently of GA signaling. Nonetheless, *procera* is a leaky mutant, the constitutive GA response conferred by the *pro* mutation is not saturated (Van Tuinen et al., 1999), and these mutant plants are responsive to GA_3_. Thus, ABA could also act upstream of GA signaling modulating GA levels. In this respect, it has been established that the stabilization of DELLAs by ABA treatments in *Arabidopsis* plants is achieved by reducing GA accumulation (Zentella et al., 2007).

**FIGURE 4:**
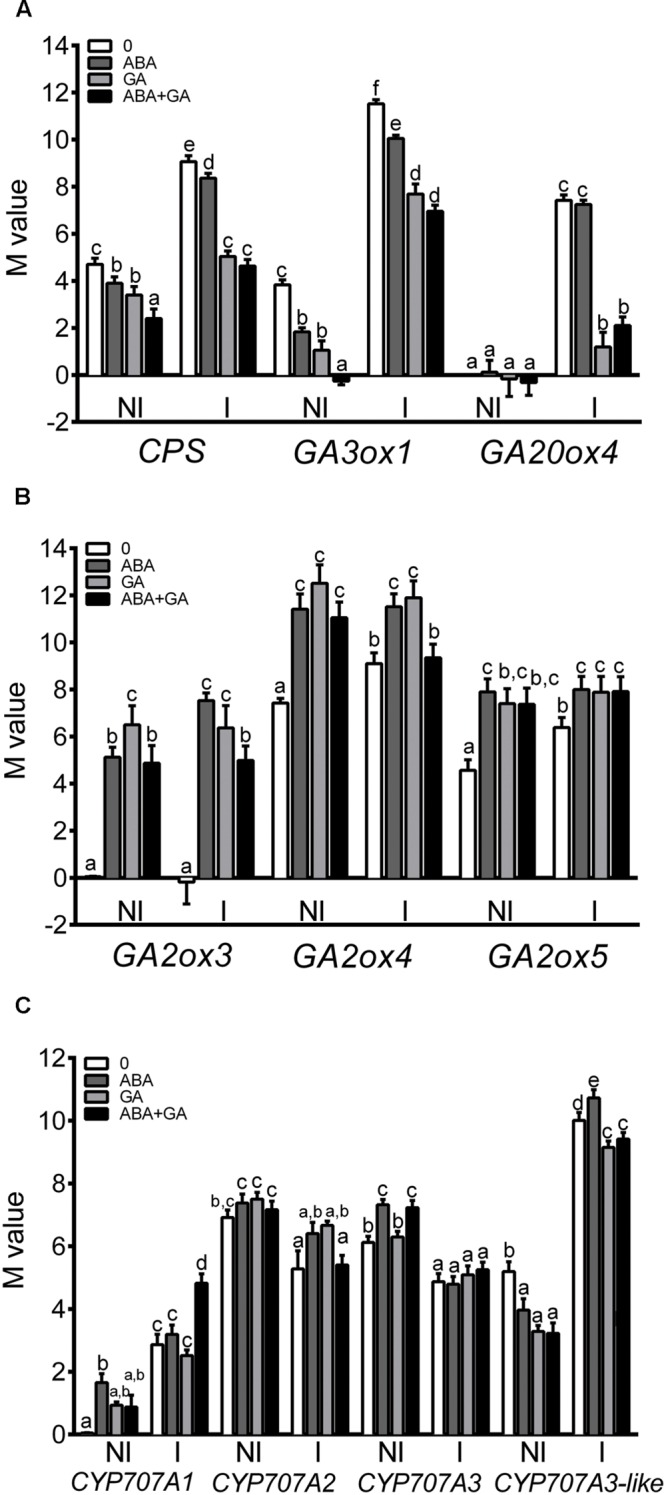
**Effect of ABA and GA_3_ applications on GA biosynthesis **(A)** GA catabolism **(B)** and ABA catabolism-related **(C)** gene expression in roots of Moneymaker tomato plants non-colonized (NI) or colonized with *R. irregularis* (I).** After 1 week of transplanting and inoculation with *R. irregularis*, a set of tomato plants (control) were treated with 0.1% ethanol solution, and three sets of plants were treated with ABA, GA_3_ and GA_3_+ABA. GA3 (5 μM) and ABA (75 μM) solutions were applied to soil twice per week. Gene expression was measured by qPCR 50 days after inoculation. qPCR data represents expression as *M*-value in gene expression relative to the biological replicate with lower expression in which the expression was designated to be 0 and all other samples were expressed relative to it. Values correspond to mean ± SE (*n* = 3). For each gene, bars with same letters are not significantly different (*P* = 0.05) according to Duncan’s multiple range test.

**FIGURE 5:**
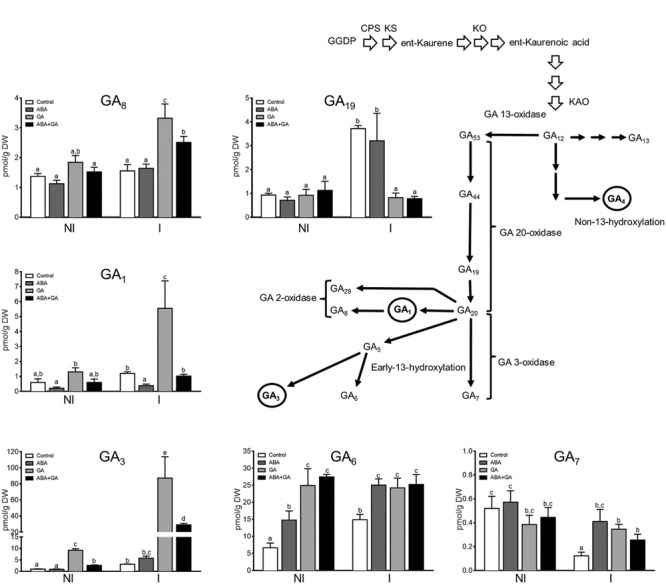
**Effect of ABA and GA_3_ applications on GAs from the non 13-hydroxylation pathway contents in roots of Moneymaker tomato plants non-colonized (NI) or colonized with *R. irregularis* (I).** After 1 week of transplanting and inoculation with *R. irregularis*, a set of tomato plants (control) were treated with 0.1% ethanol solution, and three sets of plants were treated with ABA, GA_3_, and GA_3_+ABA. GA3 (5 μM) and ABA (75 μM) solutions were applied to soil twice per week, and plants were harvested 50 days after inoculation. GA_1_, GA_3_, and GA_4_ are biologically active GAs. Reactions catalyzed by GA 20-oxidase, GA 3-oxidase and GA 2-oxidase are shown, respectively. GGDP, geranylgeranyl diphosphate; CPS, ent-copalyl diphosphate synthase; KS, ent-kaurene synthase; KO, ent-kaurene oxidase; KAO, ent-kaurenoic acid oxidase. Values correspond to mean ± SE (*n* = 3), and bars with the same letter are not significantly different (*P* = 0.05) according to Duncan’s multiple range test.

Gene transcripts accumulation as well as the metabolites concentration related to GA and ABA metabolism were measured in ABA, GA3, and ABA+GA3 treated plants. The expression patterns of GA biosynthesis-related genes (*CPS, GA30x1*, and *GA20ox4*) and GA catabolism genes (*GA2ox3, GA2ox4, and GA2ox5*) were quantified using qPCR (**Figures 4A,B**). All genes, except *GA2ox3*, exhibited positive regulation under mycorrhizal conditions. The application of exogenous ABA or GA_3_ reduced gene expression of biosynthesis-related genes (**Figure 4A**) although ABA treatment did so to a lesser extent than GA_3_ treatment. Unlike GA biosynthesis-related genes, the GA catabolism genes analyzed showed a significant and similar positive response to GA_3_ and ABA treatments, increasing their expression relative to non-treated roots (**Figure 4B**). Accordingly, the application of exogenous ABA caused a decrease in bioactive GA_1_ (the bioactive GA activated during AM) content in roots, while that of GA_6_ and GA_7_ catabolites increased (**Figure 5**). GA_6_ and GA_7_ are products of the catabolic action of GA2-/GA3-oxidases to GA_5_ and GA_20_ which are the precursors of GA_1_ and GA_3_, respectively. Interestingly, a feedback accumulation of GA_1_ and GA_3_ was observed following by exogenous GA_3_ application simultaneously with a negative effect on the level of their precursor, GA_19._ This impact was more pronounced in mycorrhizal roots than in non-mycorrhizal roots. This phenomenon is probably due to the inherent activation of the GA metabolism in mycorrhizal plants; remarkably, ABA applications interfere with this GA burst in mycorrhizal roots, implying that ABA is capable of regulating GA metabolism.

The application of GA_3_ promotes GA catabolism, measured as increases in GA_8_, GA_6_ and GA_7_ (more noticeable in mycorrhizal roots). Consistent with the increased GA-catabolic gene expression in roots treated with ABA (**Figure 4B**), the presence of exogenous ABA caused a decrease in GA_1_ content while GA_6_ and GA_7_ catabolite content increased. The application of ABA in combination with GA_3_ interferes with the positive effect on GA_1_ and GA_3_ content caused by exogenous GA_3_ applications (**Figure 5**).

Altogether, the results show that ABA attenuated the expression of GA-biosynthetic genes and increased GA-catabolic gene expression in roots, two events which should lead to a reduction in bioactive GAs.

**FIGURE 6:**
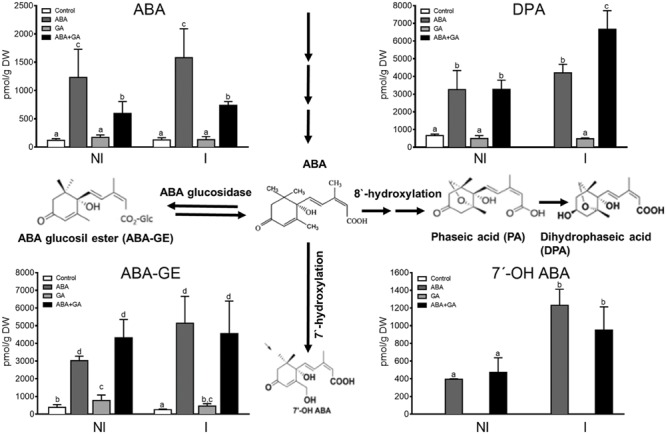
**Effect of ABA and GA_3_ applications on ABA metabolites contents in roots of Moneymaker tomato plants non-colonized (NI) or colonized with *R.* irregularis (I).** After 1 week of transplanting and inoculation with *R. irregularis*, a set of tomato plants (control) were treated with 0.1% ethanol solution, and three sets of plants were treated with ABA, GA_3_, and GA_3_+ABA. GA3 (5 μM) and ABA (75 μM) solutions were applied to soil twice per week, and plants were harvested 50 days after inoculation. Products for ABA hydroxylation and ABA glycoxidation are shown. Values correspond to mean ± SE (*n* = 3), and bars with the same letter are not significantly different (*P* = 0.05) according to Duncan’s multiple range test.

GA also regulated ABA metabolism in tomato roots. ABA-GE, free ABA and ABA catabolite levels were also determined in root samples (**Figure 6**). Exogenous applications of ABA enhance endogenous free ABA content and activate its own catabolism. GA_3_ application partially blocks the accumulation of free ABA promoting ABA glycosylation. Further, DPA and ABA-GE, which are less abundant in mycorrhizal roots than in non-mycorrhizal roots, increased after GA_3_ applications suggesting that GA_3_ enhanced the inactivation of ABA by 8′-hydroxylation in mycorrhizal roots. Meanwhile, the content of 7′-OH-ABA, which was activated after ABA treatment, was higher in mycorrhizal roots (**Figure 6**). These results as a whole suggest that GAs activate ABA catabolism and that mycorrhizal roots are more susceptible to this activation. This means that the GAs applied impact on the decrease in ABA content, a phenomenon that has previously been suggested in relation to lettuce seed germination through enhancement of ABA catabolism by gibberellins (Gonai et al., 2004).

Only small changes in gene expression related to ABA metabolism were observed following exogenous applications of the compounds (**Figure 4C**) and consequently, it is difficult to establish a correlation between the accumulation of catabolites and the expression of ABA metabolism-related genes. Thus, *CYP707A1* gene expression was activated by ABA+GA_3_ treatment mainly in mycorrhizal roots, whereas ABA and GA_3_ applications upregulated *CYP707A2* expression. *CYP707A3* gene expression was increased in non-mycorrhizal roots after ABA and ABA+GA_3_ treatments, and *CYP707A3*-like expression seems to be downregulated by all treatments except in mycorrhizal roots treated with ABA (**Figure 4C**). *NCED1* and *NCDE2* gene expression were unaffected by chemical treatments (data not shown).

### ABA/GAs Imbalance in *Sitiens:* Inhibition of GA Biosynthesis has Similar Effect than ABA Application on Mycorrhizal Recovery in the Low ABA Mutant

Arbuscular mycorrhizal development in *sitiens* plants is impaired due to ABA deficiency but is not directly associated with an increase in GA content (Supplementary Figure S3). Nevertheless this could be associated with greater responsiveness to GAs as a consequence of the ABA/GA imbalance in *sitiens* roots. Thus, we determined whether the breakdown of this imbalance affects the GA hormone pathway in this mutant which is more responsive to ABA application. Previous studies have shown that the small percentage of arbuscules observed in mycorrhizal *sitiens* plants is mainly caused by the suppression of ABA biosynthesis in these mutants and that applications of ABA resulted in normal values for arbuscule abundance in mycorrhizal *sitiens* roots (Herrera-Medina et al., 2007; Martín-Rodríguez et al., 2011).

**FIGURE 7:**
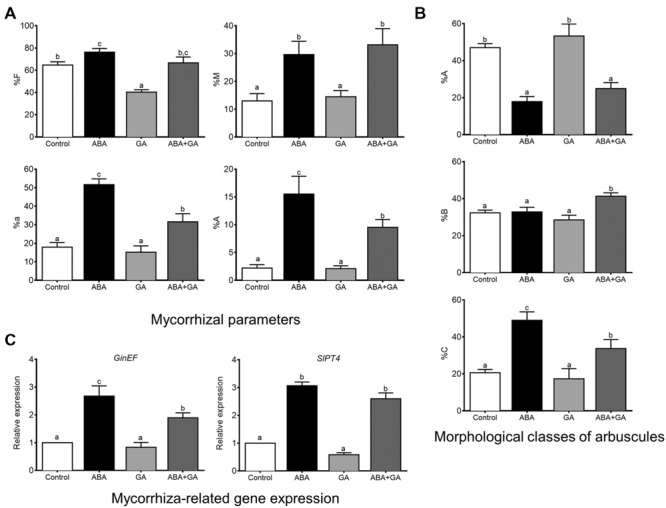
**Effect of ABA and GA_3_ applications on mycorrhizal parameters **(A)**, arbuscule distribution according to the different morphological classes **(B)** and mycorrhizal related gene expression **(C)** in roots of *sitiens* tomato plants colonized with *R. irregularis*.** After 1 week of transplanting and inoculation with *R. irregularis*, a set of *sitiens* tomato plants (control) were treated with 0.1% ethanol solution, and three sets of plants were treated with ABA, GA_3_, and GA_3_+ABA. GA_3_ (5 μM) and ABA (75 μM) solutions were applied to soil twice per week, and plants were harvested 50 days after inoculation. qPCR data represents the expression of target genes in treated plants with respect to the expression in non-treated control plants in which expression was designated as 1. Values correspond to mean ± SE (*n* = 3). Bars with same letters do not significantly differ (*P* = 0.05) according to Duncan’s multiple range test.

We studied here the effects on mycorrhization and GA metabolism of *sitiens* plants treated with different combinations of ABA and GA_3_. While the application of GA_3_ reduced %F and had no impact on the other mycorrhization parameters, the application of exogenous ABA increased all the mycorrhization parameters for *sitiens* plants, particularly %a and %A (65 and 85%, respectively; **Figure 7A**). The positive effect of ABA applications on %F, %a, and %A was attenuated when ABA was applied together with GA_3_. Nevertheless, the positive effect of ABA increasing %M occurred independently of the application of GA_3_ (**Figure 7A**). The high level of arbuscules in roots after ABA treatment probably triggers an increase in the colonization rate which also increases the intensity of colonization (%M). Contrary to what occurred with respect to arbuscule abundance, this positive effect was not eliminated when GA_3_ was applied in combination with ABA, suggesting that the positive effect of ABA on mycorrhizal intensity is independent of GAs.

The percentages of each arbuscule class (Supplementary Figure S1) for the different treatments differed greatly and confirmed the microscopic measurements of mycorrhization parameters. In *sitiens* plants, both untreated and treated with GA_3_, the majority of the arbuscules were in class A (less branched; 50 and 55%, respectively), with only 20% in class B (intermediate branched; **Figure 7B**). Conversely, in plants treated with ABA, the majority of arbuscules were in class C (full branched; 50%) with only 20% in class A (**Figure 7B**). However, with respect to plants treated with ABA in conjunction with GA_3_, the three classes of arbuscules were present in similar proportions (**Figure 7B**). Interestingly, a close linear and inverse correlation was observed between treatments in relation to the percentage of arbuscules in classes A and C (*r*^2^ = -0.95).

*GinEF* gene expression, which determines the rate of fungal colonization, increased threefold with ABA treatment and twofold in ABA+GA_3_ treated plants (**Figure 7C**), while no significant change was observed with GA_3_ treatment. The expression of *SlPT4*, a plant gene marker for arbuscules functionality, reached maximum levels in ABA and ABA+GA_3_ treated plants (**Figure 7C**). Since *SlPT4* reflects active arbuscules in tomato cells, arbuscule abundance (%a and %A) closely correlated with *SlPT4* gene expression measurement data (*r*^2^ = 0.95).

**FIGURE 8:**
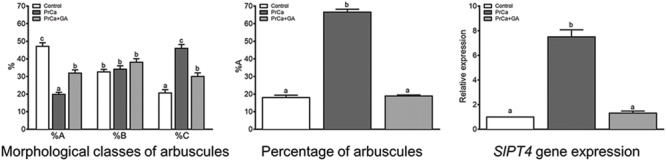
**Effect of PrCa and GA_3_ applications on arbuscular distribution according to the different morphological classes, arbuscule abundance in the whole root system (%A) and *SlPT4* gene expression in roots of *sitiens* tomato plants colonized with *R. irregularis.*** After 1 week of transplanting and inoculation with *R. irregularis*, a set of *sitiens* tomato plants (control) were treated with 0.1 % ethanol solution, and three sets of plants were treated with ABA, GA_3_, and GA_3_+ABA. GA_3_ (5 μM) and ABA (75 μM) solutions were applied to soil twice per week, and plants were harvested 50 days after inoculation. qPCR data represents the expression of target gene in treated plants with respect to the expression in non-treated control plants in which expression was designated as 1. Values correspond to mean ± SE (*n* = 3). Bars with same letters do not significantly differ (*P* = 0.05) according to Duncan’s multiple range test.

We also studied the effects on mycorrhizal recovery in *sitiens* plants due to the application of PrCa, a GA biosynthesis blocker. There was a major change in the mycorrhization parameters of the *sitiens* mutant when the PrCa was applied. In non-treated *sitiens* plants, the majority of arbuscules were in class A (near 50%) with only 20% in class C. In *sitiens* plants treated with PrCa, the majority of arbuscules were in class C (50%) with only 20% in class A (**Figure 8**). With respect to plants treated with PrCa plus GA_3_, the three classes of arbuscules were present in similar proportions (30% for class A to 35% for class B). According to these changes in arbuscule morphology, PrCa treatment increased arbuscule abundance in mycorrhizal root fragments (% a) as well as *SlPT4* gene expression. The application of GAs together with PrCa counteracted the increase in %a and *SlPT4* gene expression caused by PrCa (**Figure 8**).

As data showed, the effect on mycorrhization of ABA applications in tomato *sitiens* plants is similar to the inhibition of GA biosynthesis by PrCa applications. In these ABA-deficient tomato plants, the independent application of both ABA and PrCa increased arbuscule abundance and abundance in mycorrhized roots (%a and %A). This would indicate that there is a dependent and antagonistic relationship between ABA and GAs during AM development, although we cannot exclude that they could achieve a similar phenotype by acting independently. However, the fact that the application of GA_3_ attenuates these positive effects, suggest that the impairment of arbuscule formation by ABA deficiency is attributable to an ABA/GA imbalance in roots. Our findings on arbuscule morphology and mycorrhiza-related gene expression in *sitiens* plants treated with different compounds confirm that both ABA and PrCa have a similar positive impact on arbuscular formation in the mycorrhizal roots of ABA-deficient plants. As SlPT4 reflects plant activity in arbuscular cells, a close linear correlation exists between arbuscule abundance in mycorrhizal roots (%A), the percentage of arbuscules with many branches as well as the high intensity of trypan blue stain and *SlPT4* gene expression measurements.

As for wild-type plants, the GA pathway was clearly altered in *sitiens* plants after the application of ABA. This alteration was selective and mostly affected the 13-hydroxylation pathway (Supplementary Figure S4), decreasing the levels of bioactive GA_1_ and its precursor GA_20_ while those of GA_6_ catabolite increased (Supplementary Figure S4). Some crossover effects were detected when ABA and GA_3_ were applied jointly. The combined application of both compounds increases the concentration of GA_15_ catabolite and GA_3_ when applied together with ABA rescue the negative effect of ABA applications on GA_9_ accumulation. The application of GA_3_ in combination with ABA rescued the negative effect on GA_1_ content caused by ABA. Conversely, the application of ABA caused an increase in GA_6_ and GA_29_ compounds, which was eliminated in the presence of GA_3_. The application of ABA reduced GA_20_ content in *sitiens* roots, although there was no recovery in GA_20_ content when ABA was applied together with GA_3_. ABA applied alone or in combination with GA_3_ had a significant positive effect on the concentration of the GA_13_ metabolite (Supplementary Figure S4). The increase in GA_13_ reflects the activation of an alternative pathway to non 13-hydroxylation and 13-hydroxylation pathways of GAs biosynthesis in the presence of ABA.

The expression patterns of selected genes from the GA-biosynthetic pathway (*GA3ox1*), from the GA-catabolic pathway (*GA2ox5*) as well as GA signaling pathway (*SlDELLA*) were characterized. All three genes examined showed similar expression patterns characterized by a positive response to the application of ABA which was disabled by the presence of GA_3_ (Supplementary Figure S4).

Summarizing, ABA increased DELLA transcript accumulation, induced GA catabolism and reduced bioactive GA_1_ concentrations in mycorrhizal *sitien*s roots. Altogether, these results suggest that exogenous ABA adversely affects the GA response. These alterations are dependent on the ABA/GA balance in roots since the application of GA_3_ blocks the effects of ABA. Similarly, in *Arabidopsis* plants, it has been established that stabilization of DELLA by ABA treatments is achieved by reducing GA accumulation (Zentella et al., 2007). Some reports suggest that endogenous bioactive GA_1_ content significantly decreases with the application of PrCa, as GA biosynthesis is interrupted at later stages when GA_20_ is converted to GA_1_ (Kim et al., 2007, 2010; Kang et al., 2010). When *sitiens* plants were treated with exogenous ABA, the ABA content increased more than 10-fold in roots. Nevertheless, endogenous ABA remained at a low level following ABA+GA_3_ applications, suggesting once again that the presence of GA_3_ prevents accumulation in the roots of the exogenous free ABA applied.

## Conclusion

Arbuscule formation requires the presence of DELLA proteins and GAs negatively regulate their formation through DELLA degradation (Floss et al., 2013; Foo et al., 2013; Yu et al., 2014; Pimprikar et al., 2016). Therefore, the control of GA levels through a combination of biosynthesis and degradation provides a mechanism for regulating and fine-tuning GA levels in mycorrhizal roots (Martín-Rodríguez et al., 2015; this study) and consequently arbuscules formation. Our findings highlight a link between ABA and GAs that regulates arbuscule formation, and some of the data obtained support the hypothesis that ABA may act in part via modifying GA level to regulate AM. Accordingly, the ABA/GA hormonal balance could be regarded as an important mechanism in the regulation of symbiosis. Taken as a whole, these observations suggest that, apart from the individual role played by each of these hormones, they also perform essential overlapping functions, indicating that these isoprenoid-derived plant hormones are at the core of the network for regulating AM symbiosis. Furthermore, there are reasons to postulate that an optimum hormonal balance of ABA/GAs is essential for the regulation of others hormones such as auxin, cytokinins, strigolactone or ethylene whose participation on AM formation has also been reported recently (López-Ráez et al., 2010; Martín-Rodríguez et al., 2011; Etemadi et al., 2014; Cosme et al., 2016).

## Author Contributions

JG-G, JO, and JM-R conceived and designed the research; JM-R, RH, and TH-P performed experiments; VT, DT, and JL-M performed ABA and GAs determinations and analyzed the data; JG-G draft the manuscript. All authors have read and approved the manuscript for publication.

## Conflict of Interest Statement

The authors declare that the research was conducted in the absence of any commercial or financial relationships that could be construed as a potential conflict of interest.

## Footnotes

1 http://www.dijon.inra.fr/mychintec/Mycocalc-prg/download.html

2 http://www.bibby-scientific.com

3 http://www.beckmancoulter.com

4 http://www.waters.com

## Abbreviations

ABA: abscisic acid
ABA-GE: ABA-glucosyl ester
AM: arbuscular mycorrhiza
CPS: ent-copalyl diphosphate synthase
CYP: cytochrome P450
DPA: dihydrophaseic acid
GA: gibberellin
GAox: gibberellin oxidase
NCED: 9-*cis*-epoxy-carotenoid dioxygenase
PA: phaseic acid
PrCa: prohexadione-calcium
qRT-PCR: quantitative RT-PCR
UPLC-ESI(+)-MS/MS: ultra performance liquid chromatography-electrospray ionization-tandem mass spectrometry.
