# Supplementary material for: Gibberellin–Abscisic Acid Balances during Arbuscular Mycorrhiza Formation in Tomato
Source: Front Plant Sci. 2016 Aug 23;7:1273. doi: 10.3389/fpls.2016.01273 (PMC4993810; doi:10.3389/fpls.2016.01273)

Figure S1: Evaluation of arbuscule morphology in mycorrhizal *sitiens* plant roots. Trypan blue histochemical staining of treated *sitiens* plant roots. After one week of transplanting and inoculation with *R. irregularis*, a set of sitiens tomato plants (control) were treated with 0.1 % ethanol solution, and three sets of *sitiens* plants were treated with ABA, GA_3 and GA3+ABA. GA_3 (5mM) and ABA (75 mM) solutions were applied to soil twice per week, and plants were harvested fifty days after inoculation. To sort the arbuscules into the three morphologically different stages of arbuscule formation, at least 100 arbuscular units (arbusculated cell) were observed in root sections. Bar 50 µm.

Each arbuscular unit was placed in one of the three morphological class as folows: class A, arbuscules in formation (or degradation) with no fine branches partially occupying the plant cell; class B, arbuscules with at least some fine branches and intermediate intensity of trypan blue stain occupying almost all of the plant cell; class C, arbuscules with many branches and a high intensity of trypan blue stain occupying the whole plant cell.

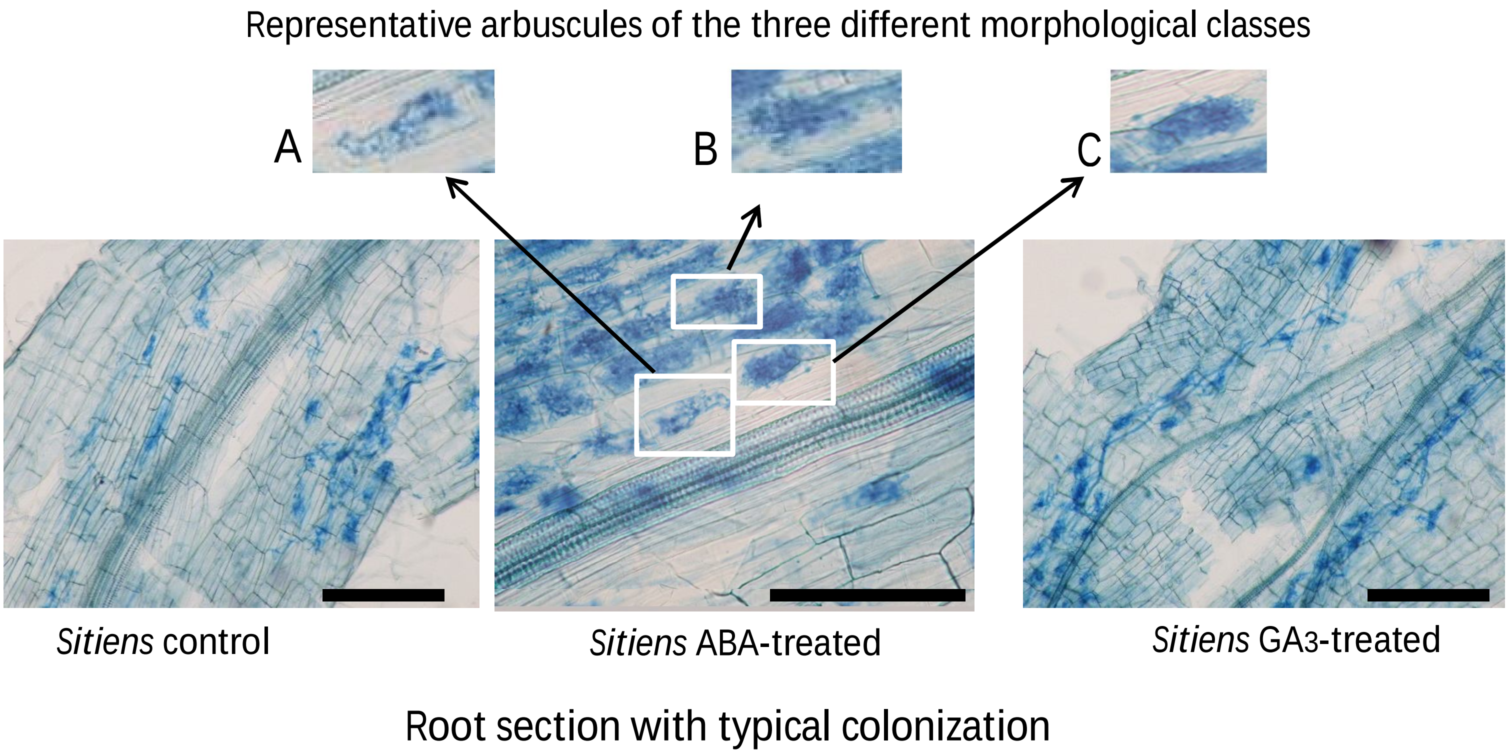

Supplement: Supplementary file 1 [file Image_1.PDF]
