# Supplementary material for: Gibberellin–Abscisic Acid Balances during Arbuscular Mycorrhiza Formation in Tomato
Source: Front Plant Sci. 2016 Aug 23;7:1273. doi: 10.3389/fpls.2016.01273 (PMC4993810; doi:10.3389/fpls.2016.01273)

Figure S2. Pie charts showing the percentage ABA metabolites in tomato roots. Levels of ABA, ABA-GE, DPA, PA, 7' OH-ABA and neo-PA were measured by UPLC-ESI(+)-MS/MS from roots of non-mycorrhizal (Non-inoculated) and mycorrhizal (Inoculated) plants forty days after inoculation with *R. irregularis*.

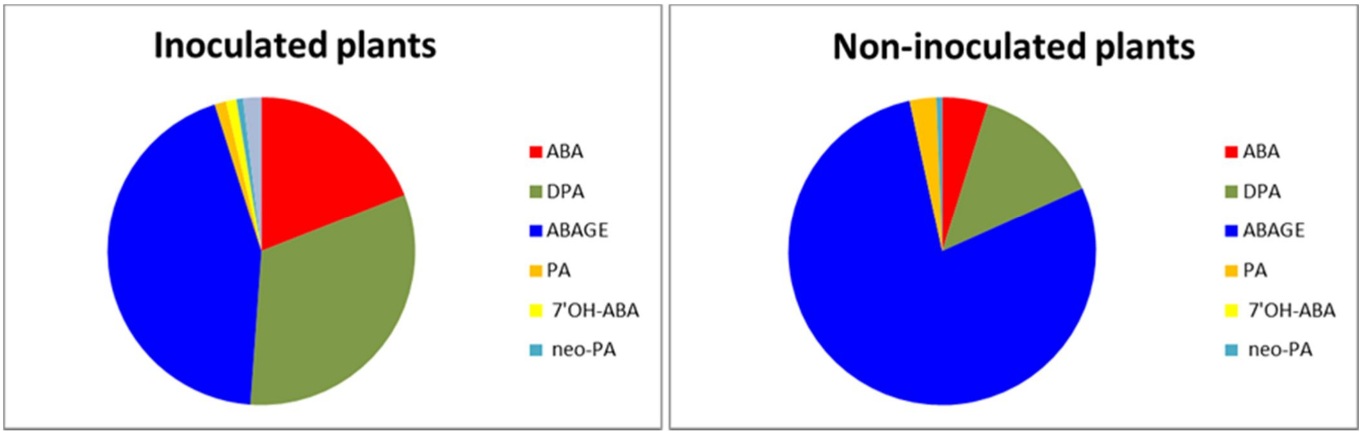

Supplement: Supplementary file 2 [file Image_2.PDF]
