# Supplementary material for: Gibberellin–Abscisic Acid Balances during Arbuscular Mycorrhiza Formation in Tomato
Source: Front Plant Sci. 2016 Aug 23;7:1273. doi: 10.3389/fpls.2016.01273 (PMC4993810; doi:10.3389/fpls.2016.01273)

Figure S3. Gibberellin content from the non 13-hydroxylation and 13-hydroxylation pathway in roots of Rheinland Ruhm (Rhe) and *sitiens* (sit) tomato plants non-colonized (NI) and colonized (I) with *R. irregularis*. After one week of transplanting, plants were inoculated with *R.irregularis*, and GAs were measured by UHPLC-MS/MS fifty days after inoculation. Values correspond to means ± SE (n=3), and bars with the same letter are not significantly different (P=0.05) according to Duncan's multiple range test.

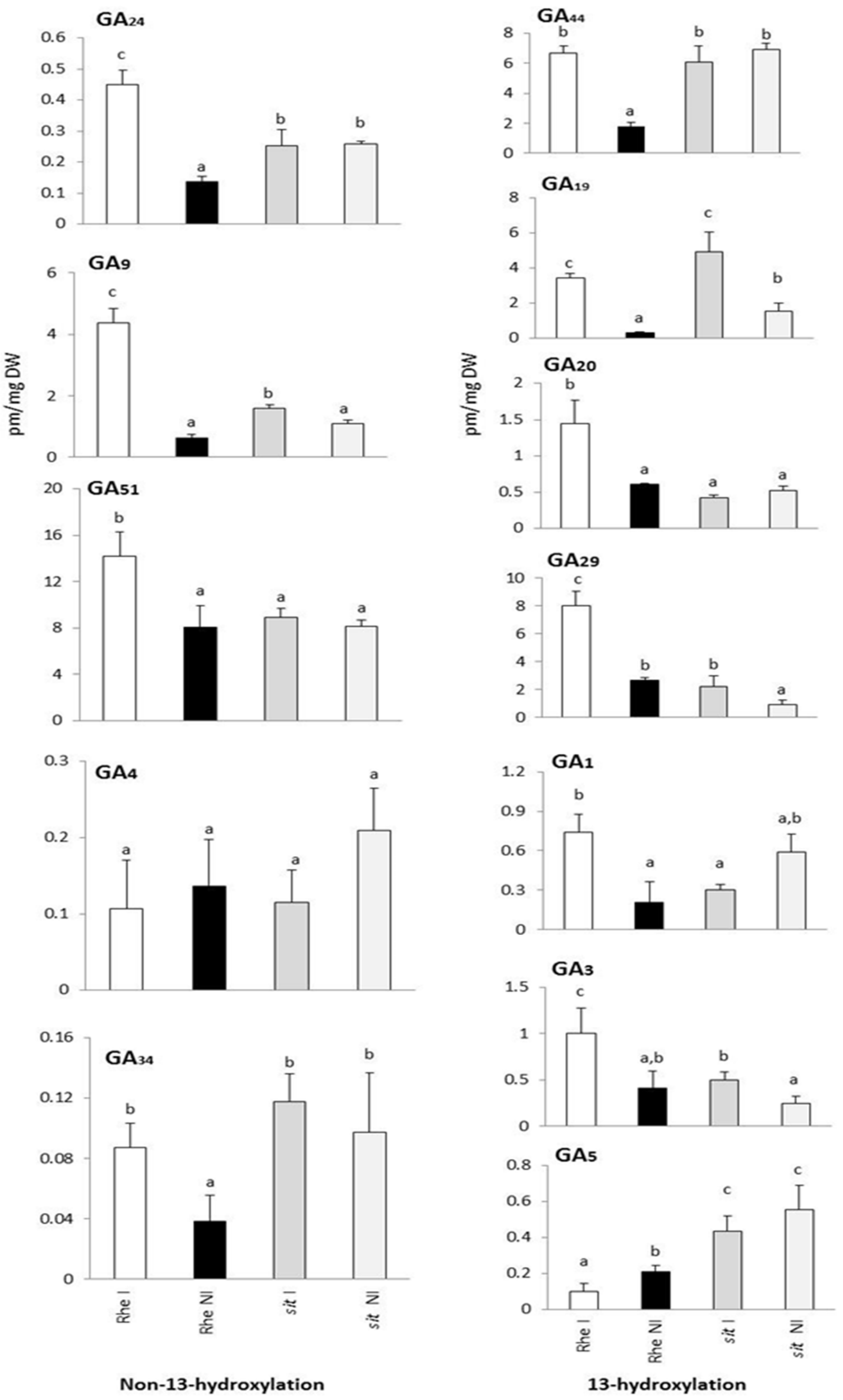

Supplement: Supplementary file 3 [file Image_3.PDF]
