# Supplementary material for: Gibberellin–Abscisic Acid Balances during Arbuscular Mycorrhiza Formation in Tomato
Source: Front Plant Sci. 2016 Aug 23;7:1273. doi: 10.3389/fpls.2016.01273 (PMC4993810; doi:10.3389/fpls.2016.01273)
Supplement: Supplementary file 4 [file Image_4.PDF]

Figure S4. Effect of ABA and GA_3 applications on gibberellins from the non 13-hydroxylation and 13-hydroxylation pathway (A) and GA pathway-related gene expression (B) in roots of *sitiens* tomato plants colonized with *R. irregularis*. After one week of transplanting and inoculation with *R. irregularis*, a set of *sitiens* tomato plants (control) were treated with 0.1 % ethanol solution, and three sets of plants were treated with ABA, GA_3 and GA_3+ABA. GA_3 (5μM) and ABA (75 μM) solutions were applied to soil twice per week, and plants were harvested fifty days after inoculation. qPCR data represents the expression of target gene in treated plants with respect to the expression in non-treated control plants in which expression was designated as 1. Values correspond to means ± SE (n=3). Bars with similar letters do not significantly differ (P=0.05) according to Duncan's multiple range test.

A

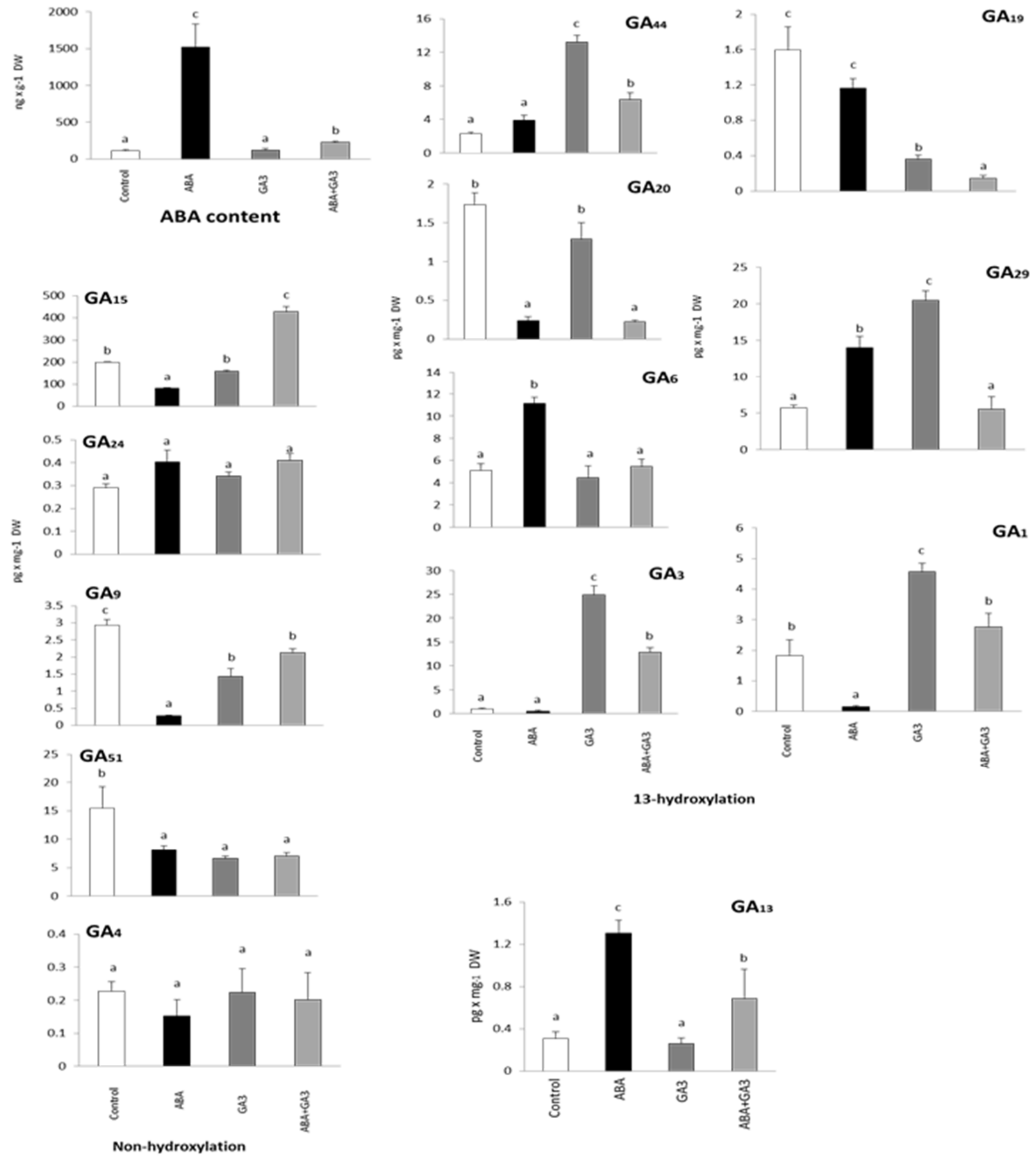

B

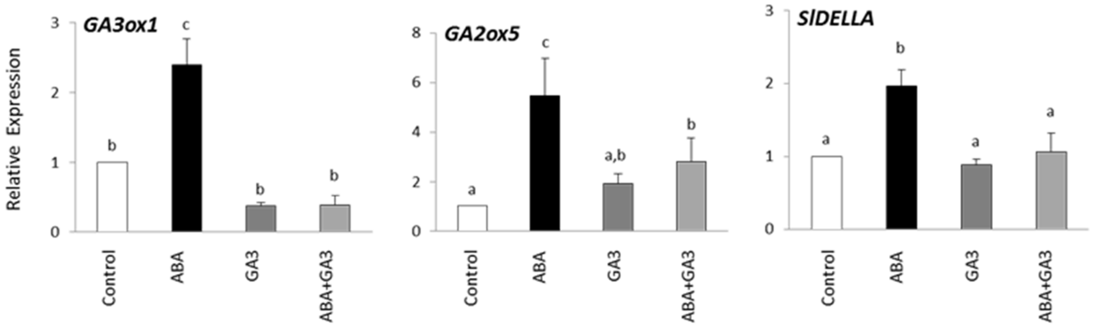

GA pathway-related gene expression
