# Supplementary material for: Gibberellin–Abscisic Acid Balances during Arbuscular Mycorrhiza Formation in Tomato
Source: Front Plant Sci. 2016 Aug 23;7:1273. doi: 10.3389/fpls.2016.01273 (PMC4993810; doi:10.3389/fpls.2016.01273)
Supplement: Supplementary file 5 [file Table_1.DOCX]

| **Primer name** | **Organism** | **Target gene** | **Primer sequence (5’- 3’)** | **Biological function** | **Reference** |
| --- | --- | --- | --- | --- | --- |
| EF-1aF | *Solanum lycopersicum* | *SlEF1α* | (5´-GGTGGCGAGCATGATTTTGA-3´) | Elongation factor 1α | García Garrido *et al.,* (2010) |
| EF-1aR |  |  | (5´-CGAGCCAACCATGGAAAACAA-3´) |  |  |
| GA3ox1-F | *S. lycopersicum* | *SlGA3ox1* | (5’-TCACTGTCCCTCCAATACCC -3’) | GA biosynthesis ( 3β-hydroxylase activity) | García Garrido *et al.,* (2010) |
| GA3ox1-R |  |  | (5’-ATCGTGTTCGGTTTACGACC -3’) |  |  |
| GA20ox1-F | *S. lycopersicum* | *SlGA20ox1* | (5’-CTCATTTCTAATGCTCATCGT -3’) | GA biosynthesis (C_20_ 0xidase) | Serrani *et al.,* (2008) |
| GA20ox1-R |  |  | (5’-TGAGATGATTCTTTCTTAGCG -3’) |  |  |
| CPS-F | *S. lycopersicum* | *SlCPS* | (5’- ATACCTAGAGCTAGCGAAATC -3’) | GA biosynthesis (terpene cyclase) | Serrani *et al.,* (2008) |
| CPS-R |  |  | (5’- ACTGCCTAAATAGTACGTAACC -3’) |  |  |
| GA2ox3-F | *S. lycopersicum* | *SlGA2ox3* | (5’- GACCCTTCTACTTTCAGCTC -3’) | GA deactivation (C_2_ oxidase) | Serrani *et al.,* (2008) |
| GA2ox3-R |  |  | (5’- AAATTGAATTGTCTTCTATCCA -3’) |  |  |
| GA2ox4-F | *S. lycopersicum* | *SlGA2ox4* | (5’- ATGGAAGGAAAAGACAGTTTA -3’) | GA deactivation (C2 oxidase) | Serrani *et al.,* (2008) |
| GA2ox4-R |  |  | (5’- CTTTTCTCAAATAGGACCAAC -3’) |  |  |
| GA2ox5-F | *S. lycopersicum* | *SlGA2ox5* | (5’- GATCACTTACCAATAATCAACAG -3’) | GA deactivation (C2 oxidase) | Serrani *et al.,* (2008) |
| GA2ox5-R |  |  | (5’- CGTCATGGTTTACGACTTTA -3’) |  |  |
| SlDELLA-F | *S. lycopersicum* | *SlDELLA* | (5'-TGATGCGACTATACTTGATATAAG-3') | GA signalling | Serrani et al., 2008 |
| SllDELA-R |  |  | (5'-GGGTTAATCTGTTTAATAGAGTTC-3') |  |  |
| SlPT4-F | *S. lycopersicum* | *SlPT4* | (5´-GAAGGGGAGCCATTTAATGTGG-3´) | AM-specific phosphate transporter | this work |
| SlPT4-R |  |  | ( 5`-ATCGCGGCTTGTTTAGCATTTCC- 3´) |  |  |
| GinEF-F | *R. irregularis* | *GinEF* | (5’- GCTATTTTGATCATTGCCGCC - 3’) | Elongation factor 1α | Benabdellah *et al.,* (2009) |
| GinEF-R |  |  | (5’-TCATTAAAACGTTCTTCCGACC-3’) |  |  |
| CYP707A1-F | *S. lycopersicum* | *SlCYP707A1* | (5´-CCCAGAGTTCTTTCCTGATCCACAA-3´) | ABA catabolism (ABA hydroxylation) | Ji *et al*., (2014) |
| CYP707A1-R |  |  | (5´-GAATGCCACTACCAGATCCTACCAC-3´) |  |  |
| CYP707A2-F | *S. lycopersicum* | *SlCYP707A2* | (5´-GCAATGAAAGCGAGGAAAGAGC-3´) |  | Nitsch *et al.,* (2009) |
| CYP707A2-R |  |  | (5´-TCGAGCTGCAAAGATGACTCC-3´) |  |  |

Table S1. Primer list

Primer list continuation

| **Primer name** | **Organism** | **Target gene** | **Primer sequence (5’- 3’)** | **Biological function** | **Reference** |
| --- | --- | --- | --- | --- | --- |
| CYP707A3-F | *S. lycopersicum* | *SlCYP707A3* | (5`-CTAAGGTGGCAAGGAGGAAGC-3´) | ABA catabolism (ABA hydroxylation) | Nitsch *et al.,* (2009) |
| CYP707A3-R |  |  | (5´-GTGTCCTGGGCAGCAAAGAG-3´) |  |  |
| CYP707A4-F | *S. lycopersicum* | *SlCYP707A4* | (5`- GAGCATTCAAACCCGAAGCC -3´) | ABA catabolism (ABA hydroxylation) | Nitsch *et al.,* (2009) |
| CYP707A4-R |  |  | (5´- AATTGTACCCTGTTTCGAGCAC -3´) |  |  |
| CYP707A3-likeF | *S. lycopersicum* | *SlCYP707A3-like* | (5`- GGAATCAACTTAGCCAAACTGG -3´) | ABA catabolism (ABA hydroxylation) | Fiorilli et al.,( 2009) |
| CYP707A3-likeR |  |  | (5´- GATACTTGTTCTTCAGCATCCTAA -3´) |  | This work |
| NCED1-F | *S. lycopersicum* | *SlNCED1* | (5`- AGGCAACAGTGAAACTTCCATCAAG -3´) | ABA biosynthesis  (oxidative cleavage of cis-epoxycarotenoid) | Ji *et al.,* (2014) |
| NCED1-R |  |  | (5´-TCCATTAAAGAGGATATTACCGGGGAC -3´) |  |  |
| NCED2-F | *S. lycopersicum* | *SlNCED2* | (5`- TGGTTTTCATGGGACATTCATTAGC -3´) | ABA biosynthesis  (oxidative cleavage of cis-epoxycarotenoid) | Ji *et al.,* (2014) |
| NCED2-R |  |  | (5´- ATCTCCCTTCTCAACTCCCTATTCC -3´) |  |  |

Benabdellah et al., 2009. GintGRX1, the first characterized glomeromycotan glutaredoxin, is a multifunctional enzyme that responds to oxidative stress. Fungal Genetics and Biology 46, 94-103.

Fiorilli et al.,2009. Global and cell-type gene expression profiles in tomato plants colonized by an arbuscular mycorrhizal fungus. New Phytologist, 184: 975–987.

García-Garrido et al., 2010. Variations in the mycorrhization characteristics in roots of wild-type and ABA-deficient tomato are accompanied by specific transcriptomic alterations. Molecular Plant-Microbe Interactions 23, 651–664

Gomez et al., 2009. Medicago truncatula and Glomus intraradices gene expression in cortical cells harboring arbuscules in the arbuscular mycorrhizal symbiosis. BMC Plant Biology 9, 10.

Ji et al., 2014. SlNCED1 and SlCYP707A2: key genes involved in ABA metabolism during tomato fruit ripening. Journal of Experimental Botany doi:10.1093/jxb/eru288

Nitsch et al., 2009. Abscisic acid levels in tomato ovaries are regulated by LeNCED1 and SlCYP707A1. Planta 229:1335–1346

Serrani et al., 2008. Auxin-induced fruit-set in tomato is mediated in part by gibberellins. The Plant Journal 56, 922–934.
